# Managing the dialysis mode for people infected with COVID-19

**DOI:** 10.1080/0886022X.2020.1782230

**Published:** 2020-06-25

**Authors:** Li Wei, Jia Wang, Zou Mei, Lu Xiang-Heng, Gui-sen Li, Jun-wu Dong, Jiong Zhang

**Affiliations:** a Department of Nephrology, Wuhan Fourth Hospital, Puai Hospital, Tongji Medical College, Huazhong University of Science and Technology, Wuhan, Hubei, China; b Department of Nephrology, University of Electronic Science and Technology, Sichuan Academy of Sciences, Sichuan Provincial People’s Hospital, Sichuan clinical Research Center for Kidney Disease, Chengdu, China; c General Medicine Center and University of Electronic Science and Technology, Sichuan Academy of Sciences, Sichuan Provincial People’s Hospital, Chengdu, China; d Department of Neurology, the first people’s Hospital of Liangshan, Xichang, China; e Queen Mary Colleges, Medical College of Nanchang University, Nanchang, China

Dear Editor,

Coronavirus Disease 2019 (COVID-19) has become a pandemic. Due to its high pathogenicity and transmissibility, health workers and hospital visitors such as patient’s companions and caregivers are prone to hospital infection, particularly the case at the Hemodialysis Center (HDC) [1,2]. This presents a major challenge to people requiring hemodialysis (HD) and those managing the process. Unfortunately, there are few reports of dialysis-related management of people undergoing HD infected with COVID-19 [3]. Therefore, we report our management of people requiring HD and who were infected with COVID-19 by conducting a retrospectively review of relevant data at a hospital in Wuhan.

## Methods

Wuhan Fourth hospital’s HDC was one of the first designated centers to receive HD patients infected with COVID-19. Diagnosis of COVID-19 were according to the guideline for diagnosis and treatment of COVID-19 (trial sixth edition) published by Chinese National Health Commission on February 18, 2020 [4]. We gathered dialysis-related data including how HDC was changed, transformation of dialysis rooms, and the impact of government guidelines. Information was obtained through the hospital’s electronic medical record system and documentation at the HDC. The study protocol was authorized and approved by the Ethics Committee of Wuhan Fourth hospital.

## Results

A total of 31 patients undergoing HD were identified. We also identified 83 workers at the HDC who interacted with the patients and 78 patients’ companions.

## The transformation of HD center

**Figure 1.**
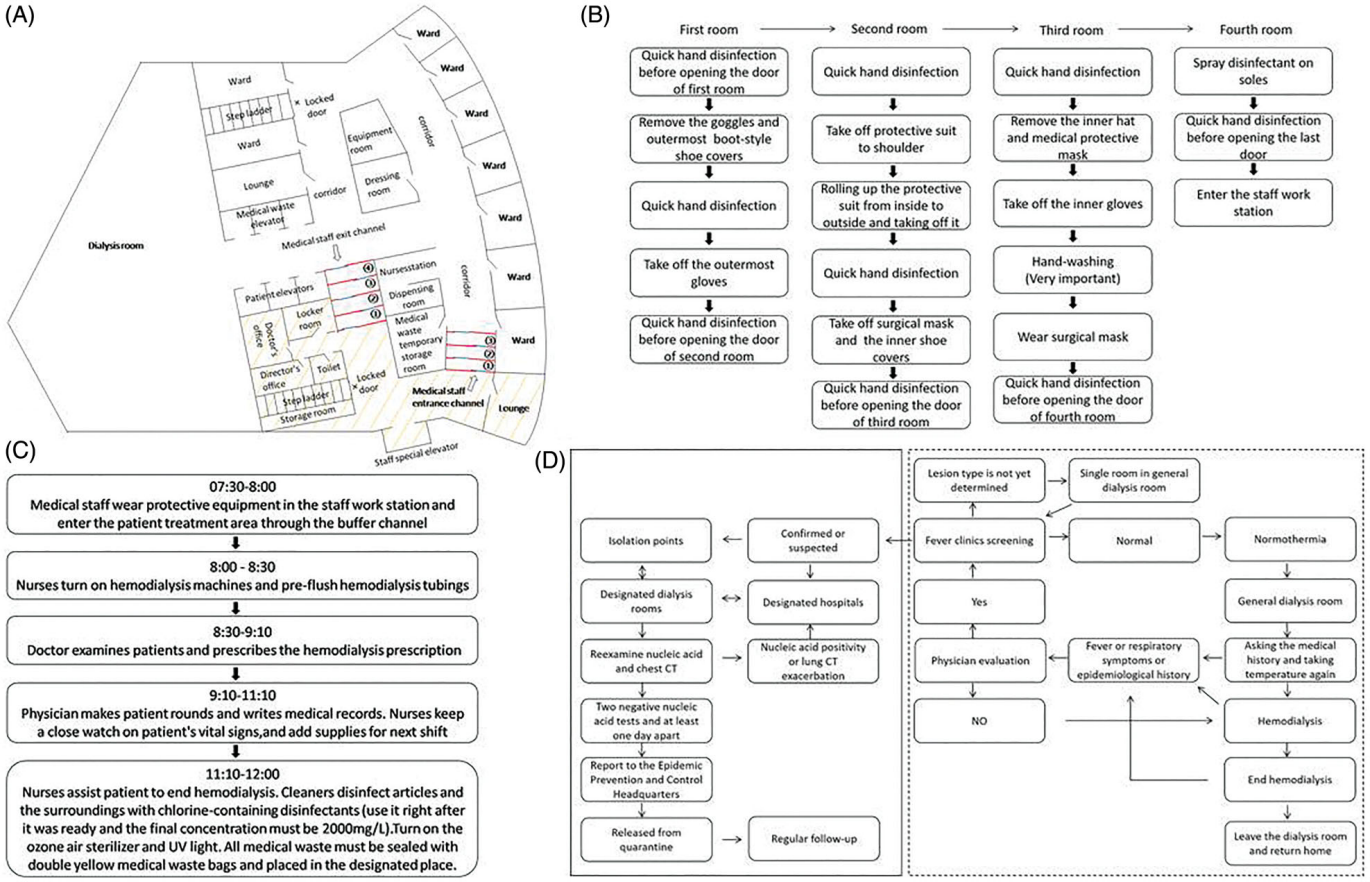
Managing HD people infected with COVID-19. (A) The layout of dialysis room; The red lines are buffer rooms, and the blue lines are the front and back doors. The yellow shaded areas are the staff work station and the rest are the patient treatment area. (B) Steps to take-off protective equipment; (C) The concrete workflow of medical staff; (D) The management process of dialysis patients. Confirmed case: nucleic acid positive; Suspected case: nucleic acid negative, but the epidemiological history or laboratory examination (high-sensitivity C-reactive protein, procalcitonin, blood routine, etc.) or lung CT conform to the characteristics of COVID-19.

After the outbreak of COVID-19, all HD patients with confirmed or suspected COVID-19 in Wuhan were transferred to designated dialysis units to effectively isolate uninfected patients. At the same time, the local government quickly built or reconstructed a lot of dialysis units designated for COVID-19 to ensure their dialysis could not be disturbed. The HDC of Gutian Branch of Wuhan Fourth Hospital was relocated and converted into two dialysis rooms. One of the specially modified dialysis rooms was used for patients with confirmed or suspected COVID-19, and the other was a general dialysis room for uninfected patients. The designated dialysis room was divided into a staff work station and a patient treatment area, where the staff work station was for employees to work, rest, and wear protective equipment, and the patient treatment area was used for HD. Two independent, non-retrograde, with three and four buffer rooms buffer channels, respectively, connecting the staff workstation and patient treatment area. The front and back doors of each buffer room were staggered from each other to avoid air convection and reduce aerosol diffusion (Figure 1(A)).

## The personnel management of HD center

### Workforce

1. The workforce consists of doctors, nurses, engineers, and other workers should strengthen COVID-19 infection training by online learning mode.
2. The staff should not gather for shift handover, business learning, case discussion, and other activities. Instead, they can use online methods to communicate by means of video conference, teleconference, and WeChat.
3. The healthcare professionals and other staff should monitor their body temperature every day. If anyone has body temperature more than 37.3°, cough or a contact with the people from epidemic area, he/she should take the initiative report and undergo medical tests promptly. Only employees who are excluded from COVID-19 infection can return to work.
4. Medical staff should wear protective equipment in the staff work station and follow the above three buffer rooms to enter the patient treatment area. After finishing work, they should gradually take off their equipment according to the prescribed steps in the four buffer rooms, and finally returned to the staff work station. Detailed steps were posted in each buffer room (Figure 1(B)).
5. Devising reasonable arrangement of personnel on duty and establishing a shift system to ensure sufficient staff on duty and avoid excessive fatigue on them. Each dialysis shift comprised of one doctor and six nurses in class.
6. The staff should have meals in different periods to avoid centralized meals. Before meal, the staff must wash hands with flowing water. During the meal, the staff should avoid talking to reduce the spread of droplets.
7. The staff should strictly follow the workflow, as shown in Figure 1(C).

### Patient’s companions

These may be family members, friends, or caregivers and they are strictly prohibited from entering the designated hospital. They will be updated about the patient’s condition by calling the medical staff on a regular basis.

### Dialysis patients

1. HD patients in the fever clinic were screened and classified as normal, confirmed or suspected COVID-19, and a temporarily uncertain lesion type [4].
2. The patients diagnosed or suspected to be COVID-19 underwent dialysis at the designated dialysis centers. Mild cases were transferred to isolation points (hotels, schools, etc.), and severe cases were transferred to designated hospitals. Patients who were temporarily uncertain the type of lesion were dialyzed in a single room in general dialysis room for 14 days, and then went to the fever clinic to screen again.
3. After careful evaluation by the expert team, patients who meet the discharge criteria (clinical symptoms improved, two negative nucleic acid tests and at least 1 day apart) would be transferred to designated isolation points for another 14-day quarantine and continue to dialysis in general dialysis rooms (Figure 1(D)).
4. Because protective clothing must be changed every 4 h, and to ensure adequate time for cleaning and disinfection, the dialysis time in the designated dialysis room was reduced to 3 h, while the dialysis time in the general dialysis room was still 4 h.

## Discussion

The “necessity”, “aggregation”, and “professionalism” of HD pose challenges to epidemic prevention and HD guarantee work. In order to avoid the spread of the epidemic and protect the work staff and patient’s companions, the HDC has performed a series of transformations, and also strengthened the management of the work staff, dialysis patients, and their companions. After the above strict management measures, the dialysis treatment of the patients was guaranteed, and there was no cross-infection in the hospital. Therefore, this study provides an effective management mode for the special population in major public health emergencies.

## Data Availability

The data that support the findings of this study are available from the corresponding author upon reasonable request.
